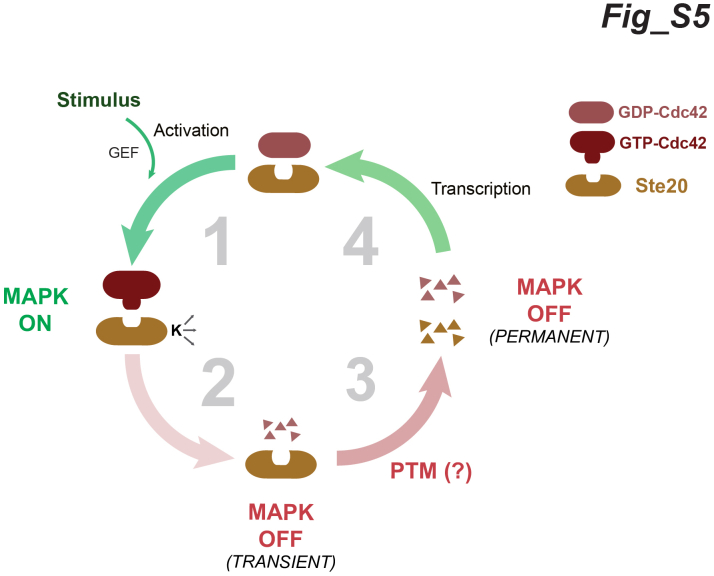# Turnover and bypass of p21-activated kinase during Cdc42-dependent MAPK signaling in yeast

**DOI:** 10.1016/j.jbc.2023.105297

**Published:** 2023-09-28

**Authors:** Beatriz González, Mahnoosh Mirzaei, Sukanya Basu, Atindra N. Pujari, Matthew D. Vandermeulen, Aditi Prabhakar, Paul J. Cullen

**Affiliations:** Department of Biological Sciences, State University of New York at Buffalo, Buffalo, New York, USA

**Keywords:** 14-3-3 proteins, Rho GTPase, p21-activated kinase, HOG, mating, basal signaling

## Abstract

Mitogen-activated protein kinase (MAPK) pathways regulate multiple cellular behaviors, including the response to stress and cell differentiation, and are highly conserved across eukaryotes. MAPK pathways can be activated by the interaction between the small GTPase Cdc42p and the p21-activated kinase (Ste20p in yeast). By studying MAPK pathway regulation in yeast, we recently found that the active conformation of Cdc42p is regulated by turnover, which impacts the activity of the pathway that regulates filamentous growth (fMAPK). Here, we show that Ste20p is regulated in a similar manner and is turned over by the 26S proteasome. This turnover did not occur when Ste20p was bound to Cdc42p, which presumably stabilized the protein to sustain MAPK pathway signaling. Although Ste20p is a major component of the fMAPK pathway, genetic approaches here identified a Ste20p-independent branch of signaling. Ste20p-independent signaling partially required the fMAPK pathway scaffold and Cdc42p-interacting protein, Bem4p, while Ste20p-dependent signaling required the 14-3-3 proteins, Bmh1p and Bmh2p. Interestingly, Ste20p-independent signaling was inhibited by one of the GTPase-activating proteins for Cdc42p, Rga1p, which unexpectedly dampened basal but not active fMAPK pathway activity. These new regulatory features of the Rho GTPase and p21-activated kinase module may extend to related pathways in other systems.

Mitogen-activated protein kinase (MAPK) pathways are evolutionary conserved modules that regulate a multiplicity of cellular responses including the response to stress (like osmotic stress), as well as cell proliferation, cell differentiation, and survival (1, 2). In response to extracellular stimuli, MAPK pathways are activated by a diverse collection of receptors and sensors that typically function at the cell surface. Once activated, sensor proteins control the activity of G-proteins, like the monomeric Rho-type GTPase Cdc42p, and effector kinases, commonly members of the p21-activated kinase (PAK) family (3). The effector module of G-proteins and PAK kinases includes three kinases in a tandem series: MAP kinase kinase kinase (MAPKKK), MAP kinase kinase (MAPKK), and MAP kinase (4, 5). One of the main functions of MAP kinases is the phosphorylation and activation of transcription factors that alter gene expression to mount a biological response. MAPK pathways can be induced by multiple stimuli, and accordingly, the core module can be ‘shared’ by different pathways to permit specific responses in different settings. Cross-talk between pathways occurs in normal settings (6, 7) but when misregulated can alter normal cellular responses and lead to diseases including cancer (2, 6, 8). Understanding how pathways are regulated and induce pathway-specific signals is critical to understanding pathway misregulation and disease in higher organisms.

One model to study MAPK pathways is the budding yeast *Saccharomyces cerevisiae*. Three of the five yeast MAPK pathways in yeast are regulated by the Rho GTPase Cdc42p and PAK kinase Ste20p [(9, 10, 11, 12) filamentous growth, mating, and HOG]. The MAPK pathway that controls filamentous growth (fMAPK) induces cell differentiation in response to nutrient (nitrogen or carbon) limitation [Fig. 1*A*, (13, 14)]. Filamentous growth is an underlying cause of virulence in plant and animal pathogens (15, 16). In *S. cerevisiae*, the fMAPK pathway is regulated by the mucin-type glycoprotein Msb2p (17), the tetra-span osmosensor Sho1p (18, 19, 20, 21), and an integral membrane protein called Opy2p (22, 23, 24), whose main function is the plasma membrane recruitment of the MAPKKK Ste11p (25). Cdc42p is activated by proteins that converge on and regulate the guanine nucleotide exchange factor (GEF) for Cdc42p, called Cdc24p. Cdc42p activity is also controlled by GTPase activating proteins (GAPs), and Rga1p is the primary GAP for Cdc42p that regulates the mating (20, 26) and fMAPK pathways (27). Among the proteins that regulate the Cdc42p module are the fMAPK scaffold Bem4p (28), the Ras-type GTPase Rsr1p (29), which mainly controls bud-site selection (30), and the polarity scaffold Bem1p (29), which regulates Cdc42p function during polarity establishment and signaling (31, 32, 33, 34).

**Figure 1:**
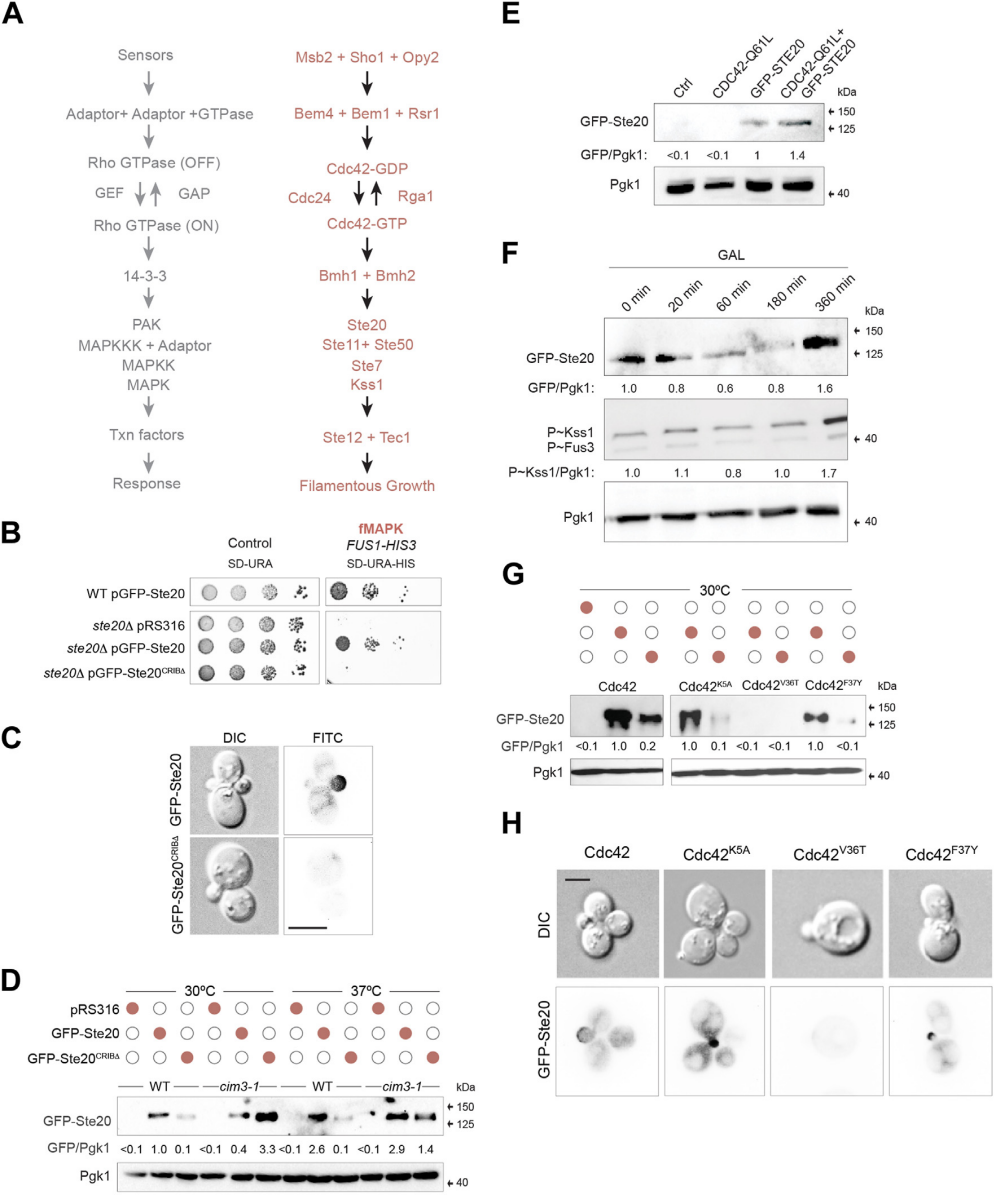
**Role of the CRIB domain of Ste20p on the localization and turnover of the protein.***A*, the fMAPK pathway. At *left*, generic names for the protein components. At *right*, names of specific proteins that regulate the fMAPK pathway. *B*, WT cells (WT, PC538, cells lack Ste4p) and cells lacking Ste20p (PC673) and expressing an empty plasmid (pRS316; PC2207), pGFP-Ste20p (PC4394), or pGFP-Ste20p^CRIBΔ^ (PC4395) were spotted onto SD-URA and SD-URA-HIS media to evaluate the activity of the *FUS1-HIS3* growth reporter. Plates were incubated for 3 days at 30 °C. *C*, localization of GFP-Ste20p and GFP-Ste20p^CRIBΔ^ in WT cells and the *ste20Δ* mutant. Strains and plasmids in panel *B* were used. Scale bar represents 5 μm. *D*, immunoblot analysis of WT cells (PC5852) and the *cim3-1* mutant (PC5851) that contain pRS316 (CTL), pGFP-Ste20p (PC4394), and pGFP-Ste20p-CRIB (PC4395). Cells were grown at 30 °C for 5 h and shifted to 37 °C for 2 h. Extracts were probed with anti-GFP antibodies to detect GFP-Ste20 and anti-Pgk1 antibodies as a loading control. Band intensities were determined for the blots shown, and ratios are reported as indicated. *E*, the level of GFP-Ste20p in cells expressing WT or a GTP-locked version of Cdc42p, GFP-Cdc42p^Q61L^. See (*D*) for details. *F*, the level of GFP-Ste20p in cells grown in galactose for the time points indicated. See (*D*) for details. *G*, immunoblots were performed with WT cells (PC538) and cells harboring *CDC42* alleles K5A, V36T, and F37Y containing the same plasmids as in (*D*). *H*, strains described in (*E*) were analyzed by fluorescence microscopy. Scale bar represents 5 μm.

The main target of the Cdc42p-Ste20p module is the MAPKKK Ste11p, which functions to phosphorylate and activate the MAPKK Ste7p, which in turn phosphorylates the MAPK Kss1p (35, 36, 37, 38). Phosphorylation of Kss1p causes activation of the protein and relief of its inhibitory functions (39), which leads to the induction of transcription factors including Ste12p (40), Tec1p (41, 42, 43, 44), Msa1p, and Msa2p (45), as well as loss of the repressive effects of negative regulators Dig1p and Dig2p (46, 47, 48). The net effect is transcriptional induction of target genes whose products bring about the filamentous cell type.

Cdc42p, Ste20p, Ste11p, and other proteins regulate the fMAPK pathway as well as the HOG and mating pathways (24, 49, 50, 51, 52). Each pathway has its own MAP kinase: Kss1p for the fMAPK pathway, Hog1p for HOG pathway, and Fus3p for the mating pathway (53). Kss1p and Fus3p are both phosphorylated during mating to modulate the mating response (54). Each pathway also contains a pathway-specific scaffold. The well-studied scaffold Ste5p is an adaptor for the mating pathway (55, 56, 57, 58, 59). Ste5p is required for the phosphorylation of Fus3p (60). Ste5p binds to the MAPK Fus3p and catalytically unlocks the protein, making it available for phosphorylation by Ste7p (61). Pbs2p [also the MAPKK (62)] and Ahk1p (63) are scaffolds for the HOG pathway, and Bem4p is a scaffold for the fMAPK pathway (28). Other proteins also regulate the fMAPK pathway, such as the 14-3-3 proteins Bmh1p and Bmh2p (64), although whether these proteins play a role in pathway specification remains incompletely explored.

We previously showed that Cdc42p can be degraded to modulate the activity of the fMAPK pathway (65). By further examining potential regulatory mechanisms surrounding the Cdc42p-PAK module in this pathway, we found that Ste20p is also degraded by the 26S proteasome. Moreover, binding of Ste20p to Cdc42p stabilizes Ste20p protein levels. We propose that activation of the fMAPK pathway leads to turnover of Cdc42p and Ste20p to attenuate MAPK pathway signaling. We also identified a Ste20p-independent branch of the fMAPK pathway. The Ste20p-dependent branch required Bmh1p and Bmh2p, whereas the Ste20p-independent branch involved the fMAPK pathway adaptor Bem4p. By exploring other Cdc42p-binding proteins, we identified a specific role for the GAP Rga1p in basal MAPK signaling. These new regulatory features may extend to other Rho-dependent signaling pathways in eukaryotic organisms.

## Results

### Ste20p is turned over in the 26S proteasome and is stabilized by its interaction with Cdc42p

The active or GTP-bound conformation of Cdc42p interacts with the PAK kinase Ste20p to regulate the fMAPK pathway (Fig. 1*A*). In the N terminus of the protein, Ste20p contains a characteristic Cdc42p- and Rac-interactive binding motif (*CRIB*) that when bound to Cdc42p relieves auto-inhibition of the kinase domain of the protein (35, 36, 66, 67). The activity of the fMAPK pathway can be evaluated by transcriptional reporters whose activity provides a readout of pathway activity. A transcriptional reporter that normally functions in mating (*FUS1-HIS3*) provides a readout of fMAPK pathway activity in cells lacking an intact mating pathway [*ste4*, (17)]. The WT and a strain lacking Ste20p (*ste20*Δ) were examined containing a control plasmid (pRS316, URA3-marked) or plasmids containing genes encoding GFP-fusions to Ste20p (pGFP-Ste20p) or a version of Ste20p lacking the CRIB (334–369Δ) domain (pGFP-Ste20p^CRIBΔ^). Based on this reporter, cells lacking Ste20p were defective for induction of the *FUS1-HIS3* growth reporter and failed to grow on SD-URA-HIS media (Fig. 1*B*, *ste20*Δ pRS316). As expected, introduction of pGFP-Ste20p restored MAPK pathway activity and growth to the *ste20*Δ mutant. However, pGFP-Ste20p^CRIBΔ^ was defective for fMAPK pathway activity (Fig. 1*B*). These results support published results (35, 36), indicating that Ste20p interacts with Cdc42p by its CRIB domain to induce the fMAPK pathway.

Cdc42p is also required for the localization of Ste20p in buds and presumptive bud sites (66, 67, 68). In WT cells, GFP-Ste20p was found in buds by fluorescence microscopy (Fig. 1*C*), whereas GFP-Ste20p^CRIBΔ^ showed a diffuse pattern (Fig. 1*C*). Furthermore, immunoblot analysis using antibodies to the GFP epitope showed that GFP-Ste20p^CRIBΔ^ was present at lower levels than GFP-Ste20p (Fig. 1*D*, WT, 30 °C). This finding indicates that the CRIB domain may be required for normal levels of Ste20p in the cell.

Protein levels can be impacted by turnover, such as by the ubiquitination and degradation of proteins by the 26S proteasome (69). To test Ste20p protein levels, the *cim3-1* mutant was examined, which contains a conditional mutation in the gene encoding the proteasomal ATPase Rpt6p and which is required for 26S proteasome function (70). In the *cim3-1* mutant, the level of GFP-Ste20p^CRIBΔ^ was higher than seen in WT cells (Fig. 1*D*, *cim3-1*, 30 °C and 37 °C). The fact that GFP-Ste20p^CRIBΔ^ levels were elevated at 30 °C indicates that a partial defect in proteasome function is sufficient to restore normal levels of Ste20p. By comparison, the level of GFP-Ste20p was not impacted in the *cim3-1* mutant at 30 °C or 37 °C. This may be because WT Ste20p is a stable protein (71, 72), and changes in its levels may not be evident at the time period examined. These results reveal a new aspect of Ste20p regulation, by turnover of a version of the protein that is defective for interaction with Cdc42p in the 26S proteasome.

Ste20p lacking its CRIB domain might be unstable and degraded because the protein is misfolded. Alternatively, Ste20p lacking its CRIB domain might be turned over due to its inability to bind Cdc42p. In line with this possibility, a version of Cdc42p that mimics the GTP-locked version of the protein, Cdc42p^Q61L^ (73), which is expected to constitutively bind Ste20p, showed higher levels of Ste20p than cells expressing a WT copy of Cdc42p (Fig. 1*E*). Similarly, growth of cells in galactose, which causes an increase in the levels of GTP-bound Cdc42p (74), caused an increase in GFP-Ste20p levels (Fig. 1*F*). These results indicate that the level of Ste20p in the cell is impacted by the level of active Cdc42p.

To further explore this possibility, cells expressing versions of Cdc42p that were compromised for specific functions were examined. In particular, Cdc42p^V36T^ and Cdc42p^F37Y^ contain amino acid substitutions in the switch I domain, which is required for binding proteins with CRIB motifs, including Ste20p and other effectors including Cla4p, Gic1p, and Gic2p (75, 76). As controls, WT Cdc42p was compared to a version that contains an amino acid change in a domain unrelated to its interaction with effector proteins (Cdc42p^K5A^). Compared to controls (Cdc42 and Cdc42^K5A^), cells expressing Cdc42p^V36T^ expressed as the sole copy in the cell showed reduced levels of GFP-Ste20p by immunoblot analysis (Fig. 1*G*, Cdc42p^V36T^). By fluorescence microscopy, the level of GFP-Ste20p was also reduced in cells expressing Cdc42p^V36T^ (Fig. 1*H*, Cdc42p^V36T^). Cdc42p^F37Y^ also showed reduced levels of GFP-Ste20p but not to the same degree as Cdc42p^V36T^ (Fig. 1*G*) and showed a normal localization pattern (Fig. 1*H*). This may be because Cdc42p^V36T^ exhibits a more severe defect in fMAPK pathway activity (29), and interaction with Cdc42p (77), than Cdc42p^F37Y^. These results support the idea that the interaction between Cdc42p and Ste20p is important for maintaining normal levels of Ste20p in the cell.

**Figure S1:**
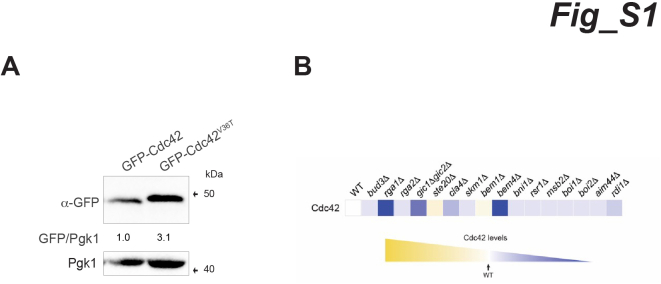


These observations led us to ask whether Ste20p may play a role in regulating Cdc42p levels as well. The active or GTP-bound conformation of Cdc42p is preferentially turned over in the proteasome compared to the WT conformation, which may result from interaction with effector proteins like Ste20p (65). To test this possibility, the level of the Cdc42p^V36T^ protein was also examined. GFP-Cdc42p^V36T^ was present in the cell at higher levels than WT GFP-Cdc42p (Fig. S1*A*). The level of Cdc42p was also higher in cells lacking Ste20p [Fig. S1*B*, heat map generated using data from (65)]. This result could be interpreted to mean that the interaction of Cdc42p with effector proteins like Ste20p results in turnover of the protein. The turnover regulation of a Rho GTPase and PAK kinase module adds a new layer of regulation to MAPK pathway signaling.

### A Cdc42p-dependent branch of the fMAPK pathway functions outside of Ste20p and requires the adaptor protein Bem4p

**Figure 2:**
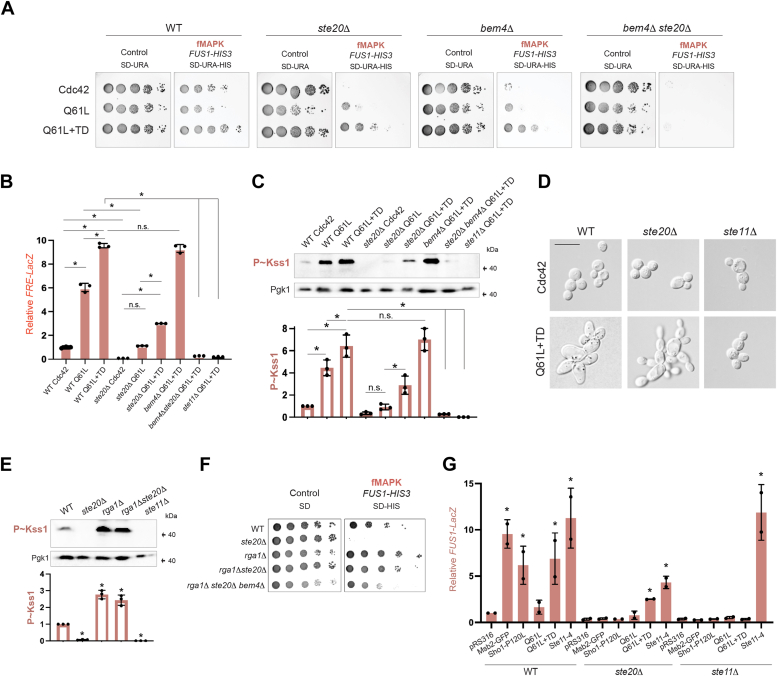
**fMAPK pathway activity independent of Ste20p.***A*, WT cells and the indicated mutants expressing pGFP-Cdc42p (Cdc42; PC6454), pGFP-Cdc42p^Q61L^ (Q61L, PC7458) or pGFP-Cdc42p^Q61L+TD^ (Q61L+TD, PC7654) were grown on SD-URA or SD-URA-HIS media. See Figure 1*B* for details. *B*, β-galactosidase assay of the *FRE-LacZ* transcriptional reporter to analyze fMAPK pathway activity. WT cells (WT; 6810) and the *ste20*Δ (PC7772) mutant expressing same plasmids explained in panel *A*. Cells in panel *B* were grown for 5 h in SD-URA-LEU media. Values show the relative transcriptional activation compared to WT cells expressing Cdc42p. Error bars represent the SD from three biological replicates (n =3). Data were analyzed by one-way ANOVA, and *p* values (*asterisk* < 0.05) were obtained using Tukey’s multiple comparison test. *C*, levels of P∼Kss1p of same strains as described in (*B*) grown for 6 h in SD-URA media. Error bars represent the SD from three biological replicates (n= 3). Data were analyzed by one-way ANOVA, and *p* values (*asterisk* < 0.05) were obtained using Tukey’s multiple comparison test. *D*, microscopic examination of WT cells, and the s*te20*Δ and *ste11*Δ mutans expressing WT Cdc42p (Cdc42) and Cdc42p^Q61L+TD^ (Q61L+TD) grown for 24 h on SD-URA media. Scale bar represents 15 μm. *E*, levels of P∼Kss1p of WT strains and the indicated mutants grown for 5 h in SD-URA media. Error bars represent SD from three biological replicates (n = 3). Data were analyzed by one-way ANOVA, and *p* values (*asterisk* < 0.05) were obtained using Tukey’s multiple comparison test. *F*, WT (PC538) cells and the *rga1*Δ (PC3391), *ste20*Δ (PC673), *rga1*Δ *ste20*Δ (PC7777), and *rga1*Δ *ste20*Δ *bem4*Δ (PC7886) mutants were grown on SD or SD-HIS media to assess the activity of *FUS1-HIS3* growth reporter. See Figure 1*B* for details. *G*, WT cells and the s*te20*Δ and *ste11*Δ mutans expressing pRS316 (PC2207), Msb2-GFP (PC1696), Sho1p^P120L^ (PC1715), Cdc42p^Q61L^, Cdc42p^Q61L+TD^, or Ste11-4 (PC1441) were grown in SGAL-URA media to evaluate the activity of the *FUS1-LacZ* reporter. Error bars represent SD from two biological replicates (n = 2). Data were analyzed by one-way ANOVA, and *p* values (asterisk < 0.05) were obtained using Tukey’s multiple comparison test. TD, turnover defective.

**Figure S2:**
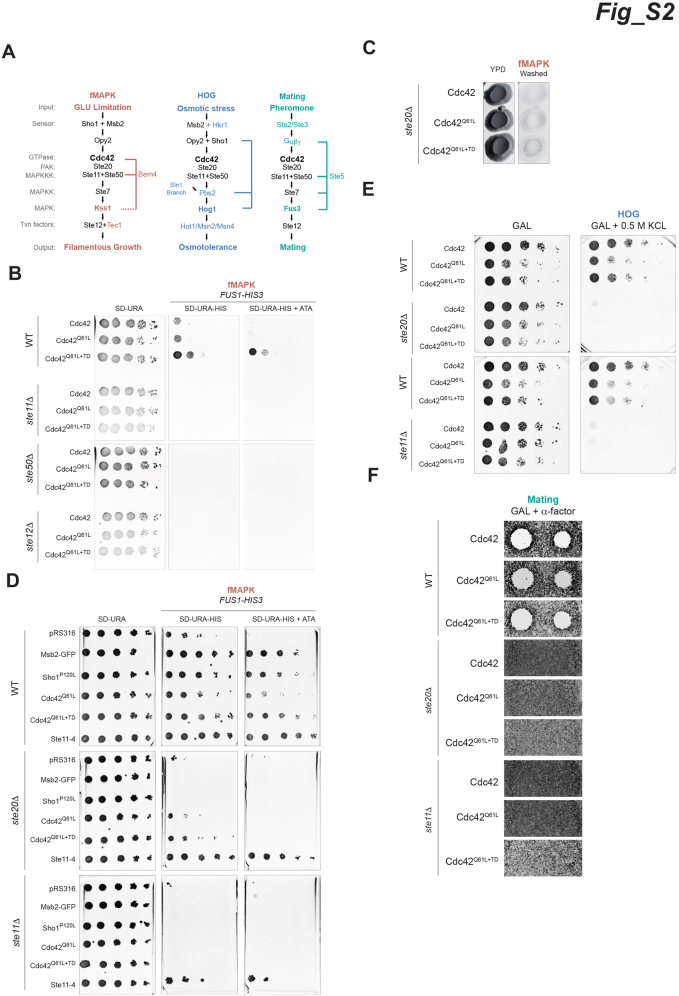


We previously identified a turnover-defective (TD) and GTP-locked (Q61L) version of Cdc42p that hyperactivates the fMAPK pathway [Cdc42p^Q61l+TD^ (65)]. This tool allowed evaluation of Cdc42p-dependent MAPK pathways (mating, filamentous growth, and HOG, Fig. S2*A*) in different genetic contexts. As reported (65), Cdc42p^Q61L+TD^ induced the fMAPK pathway to slightly higher levels than GTP-locked Cdc42p, based on the *FUS1-HIS3* growth reporter (Fig. 2*A*). Surprisingly, Cdc42p^Q61L+TD^, and to some degree Cdc42p^Q61L^ (73), induced the fMAPK pathway in cells lacking Ste20p (Fig. 2*A*, *ste20*Δ). Because this observation was unexpected, the activity of the fMAPK pathway was assessed by additional reporters of the fMAPK pathway. Another fMAPK pathway–dependent transcriptional reporter, *FRE-lacZ* (78), showed that Cdc42p^Q61L+TD^ partially bypassed the requirement for Ste20p (Fig. 2*B*, *ste20*Δ). Cdc42p^Q61L^ appears to strongly hyperactivate the *FRE-lacZ* reporter compared to *FUS1-HIS3* (Q61L compare Figure 2, *A* and *B*); however, use of the competitive inhibitor 3-amino-1,2,4 triazole showed that the reporters behaved similarly (Fig. S2*B*). Cdc42p^Q61L+TD^ did not bypass the requirement for the MAPKKK Ste11p (Figs. 2*B* and S2*B*, ste11Δ), indicating that Cdc42p signaling was independent of Ste20p and dependent on Ste11p. The Ste11p-interacting protein Ste50p (79, 80, 81) and transcription factor Ste12p (82) were also required for signaling induced by Cdc42p^Q61L+TD^ (Fig. S2*B*). The activity of the fMAPK pathway was also measured by phosphorylation of the MAP kinase, Kss1p, which showed the same results (Fig. 2*C*, see quantitation below the graph). The level of P∼Kss1p in cells lacking Ste20p was reduced compared to WT cells (Fig. 2*C*, WT Cdc42^Q61L+TD^), which as seen for *FRE-lacZ,* is indicative of a partial bypass. Activation of the fMAPK pathway induces the formation of elongated cells capable of invasive/pseudohyphal growth. Cdc42p^Q61L+TD^ induced cell elongation in cells lacking Ste20p, which was dependent on Ste11p (Fig. 2*D*). The fMAPK pathway also induces invasive growth that can be examined by the plate-washing assay (14). Cells lacking Ste20p showed detectable invasive growth when expressing Cdc42p^Q61L^ and Cdc42p^Q61L+TD^ (Fig. S2*C*). These observations together indicate that Cdc42p regulates a Ste20p-independent branch of the fMAPK pathway.

As a separate test to show Cdc42p-dependent signaling that is independent of Ste20p, we examined cells lacking Rga1p, which is the main GAP that functions in the fMAPK pathway (27). Cells lacking Rga1p show higher levels of GTP-Cdc42p (83) and would be expected to show elevated levels of fMAPK pathway activity. Consistent with this idea, the *rga1*Δ mutant induced higher levels of P∼Kss1p than seen in WT cells (Fig. 2*E*, *rga1*Δ). Cells lacking Rga1p and Ste20p showed similar levels of P∼Kss1p than cells lacking Rga1p alone (Fig. 2*E*, *rga1*Δ *ste20*Δ). The *rga1*Δ *ste20*Δ double mutant also rescued the signaling defect of the *ste20*Δ mutant based on the *FUS1-HIS3* reporter (Fig. 2*F*). Collectively, these experiments support the idea that Cdc42p regulates a Ste20p-independent branch of the fMAPK pathway. Further support comes from the fact that induction of the fMAPK pathway in WT cells by newly identified stimuli can induce signaling in cells lacking Ste20p (84).

We next sought to identify factors that mediate Ste20p-independent signaling. A good candidate was the adaptor protein Bem4p, which interacts with Cdc42p and Ste11p (28, 29). Cdc42p^Q61L+TD^ did not bypass the signaling defect of the *ste20*Δ *bem4*Δ double mutant by *FUS1-LacZ* (Fig. 2*A*), *FRE-LacZ* (Fig. 2*B*), or P∼Kss1p analysis (Fig. 2*C*). As previously reported (65), Cdc42p^Q61L+TD^ bypassed the signaling defect of the *bem4*Δ single mutant (Fig. 2, *A*–*C*). *FUS1-HIS3* reporter activity in the *rga1*Δ *ste20*Δ double mutant was also somewhat reduced by the deletion of *BEM4* (Fig. 2*F*, *rga1*Δ *ste20*Δ *bem4*Δ). However, there was some signaling in the triple mutant, which indicates that at least one other protein is involved in Ste20p-independent signaling in some contexts. Therefore, Cdc42p induces Ste20p-independent signaling at least partly through Bem4p.

We next tested whether fMAPK pathway components act preferentially in the Ste20-dependent branch. Hyperactive versions of fMAPK pathway sensors Msb2p [GFP-Msb2, (19)] and Sho1p [Sho1p^P120L^, (85)] were tested and did not bypass the requirement for Ste20p in the fMAPK pathway, based on *FUS1-lacZ* (Fig. 2*G*) and *FUS1-HIS3* analysis (Fig. S2*D*). As expected, a plasmid expressing the hyperactive allele of the MAPKKK *STE11-4* did bypass the need for Ste20p. Therefore, Msb2p and Sho1p may selectively regulate the Ste20p branch of the fMAPK pathway. To summarize, the fMAPK pathway is composed of Ste20p-dependent and Ste20p-independent branches that are regulated by different combinations of proteins.

Because Ste20p is a common component shared between three Cdc42p-dependent MAPK pathways (fMAPK, mating, and HOG, Fig. S2*A*), we next tested whether Cdc42p^Q61L+TD^ bypassed the requirement for Ste20p in the HOG and mating pathways. Cdc42p^Q61L+TD^ did not bypass the requirement for Ste20p in the HOG (Fig. S2*E*) or mating pathways (Fig. S2*F*). Therefore, Cdc42p^Q61L+TD^ may preferentially stimulate the Ste20p-independent branch of the fMAPK pathway.

### 14-3-3 proteins Bmh1p and Bmh2p regulate the Ste20p branch of the fMAPK pathway

**Figure 3:**
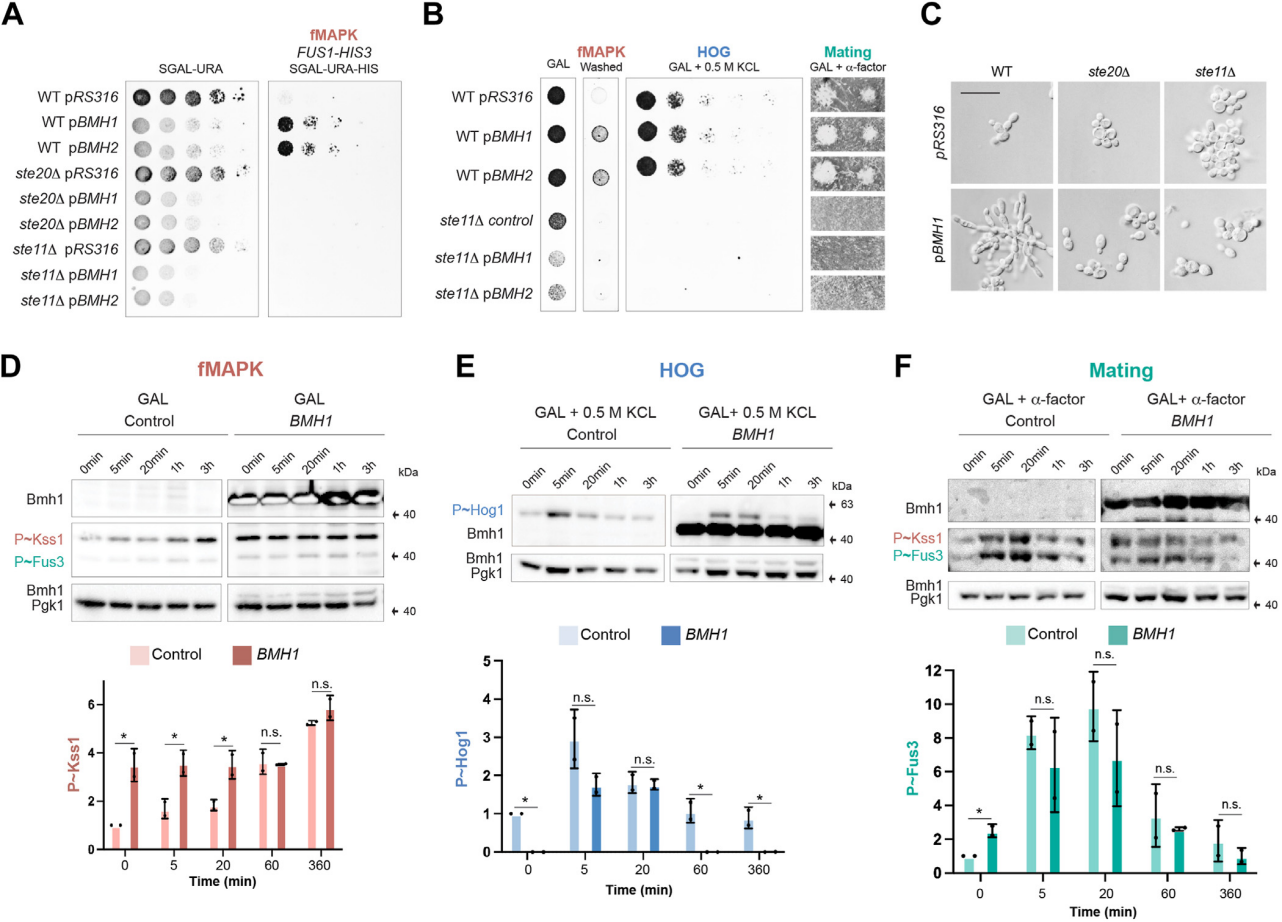
**The impact of Bmh1p and Bmh2p on fMAPK, HOG, and mating pathway activity.***A*, WT cells (PC538) and the *ste20*Δ (PC673) and *ste11*Δ (PC3862) mutants expressing pRS316, pP_*GAL1*_-BMH1, or pP_*GAL1*_-BMH2 (90) were grown on SGAL media to study the activity of the *FUS1-HIS3* reporter. See Figure 1*B* for details. *B*, WT cells (PC6810) and the *ste20*Δ (PC7772) and *ste11*Δ (PC6604 mutants) in the *ssk1*Δ background expressing same plasmids as described in panel 3A were grown on YEPGAL (Gal), YEPGAL supplemented with 0.5 M KCl, or spread onto YEPGAL supplemented with 6 μM or 1.6 μM of α-factor. *C*, microscopic examination of same cells described in panel *A* grown for 6 h in SGAL media. Scale bar represents 50 μm. For fMAPK, the plate was washed in a stream of water and photographed. *D*, the *ssk1*Δ mutant expressing pRS316 (control) or pP_*GAL1*_-BMH1 was grown during the indicated time points in YEPGAL media. Immunoblots were probed with p44/42 antibodies to detect P∼Kss1p and P∼Fus3p and with α-Pgk1p antibodies. Error bars represents SD from two biological replicates (n = 2). Data were analyzed by one-way ANOVA, and *p* values (*asterisk* < 0.05) were obtained using Tukey’s multiple comparison test. *E*, the *ssk1*Δ mutant expressing pRS316 (control) or pP_*GAL1*_-BMH1 was grown during the indicated time points in YEPGAL media supplemented with 0.5 m KCl. Immunoblots were probed with p38 antibodies to detect P∼Hog1p and with α-Pgk1 antibodies. Data were analyzed as described in panel *D*. *F*, the *ssk1*Δ mutant expressing pRS316 (control) or pP_*GAL1*_-BMH1 was grown during the indicated time points in YEPGAL media supplemented with 1.6 μM of α-factor. See (*D*) for details. Data were analyzed as described in (*D*).

**Figure S3:**
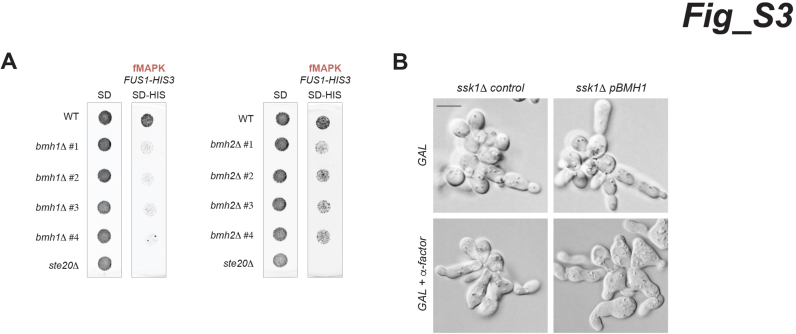


Bmh1p and Bmh2p are members of the 14-3-3 family of proteins (86) that were previously identified as Ste20p-binding proteins that also regulate the fMAPK pathway (64). *BMH1* and *BMH2* were also identified in our laboratory in a screen for genes that when overexpressed stimulate fMAPK pathway activity (87). To confirm a role for Bmh1p and Bmh2p proteins in regulating the fMAPK pathway, cells containing plasmids overexpressing the *BMH1* or *BMH2* genes were examined by the *FUS1-HIS3* reporter. Based on this test, overexpression of the *BMH* genes stimulated the fMAPK pathway (Fig. 3*A*). Overexpression of these genes also induced a growth defect, which did not require an intact fMAPK pathway (Fig. 3*A*, *ste11*Δ). Similarly, deletion of the *BMH1* or *BMH2* genes caused a decrease in *FUS1-HIS3* activity (Fig. S3*A*). We were unsuccessful in disrupting both genes, which is lethal in some strain backgrounds (88, 89) and which has previously been shown to cause a defect in filamentous growth (64). Overexpression of *BMH1* or *BMH2* also induced invasive growth (Fig. 3*B*, Washed), and overexpression of *BMH1* induced an elongated cell morphology by microscopy (Fig. 3*C*, Bmh1p and Bmh2p behaved similarly). Overexpression of *BMH1* and *BMH2* did not induce the *FUS1-HIS3* reporter in cells lacking Ste20p or Ste11p (Fig. 3*A*). Similarly, the filamentous cell morphology induced by *BMH1* was dependent on Ste20p and Ste11p (Fig. 3*C*). These results indicate that the Bmh proteins function in the Ste20p branch of the fMAPK pathway.

The Bmh1p and Bmh2p proteins might specifically regulate the fMAPK pathway or function as general regulators of Cdc42p-dependent MAPK pathways that share components. Overexpression of *BMH1* or *BMH2* did not influence the growth of cells in media supplemented with salt (Fig. 3*B*, 0.5 M KCl) or impact mating, based on sensitivity to pheromone (Fig. 3*B*, GAL + α-factor). These results indicate that the Bmh1p and Bmh2p proteins do not play a major role in regulating the HOG or mating pathways. The phosphorylation of the MAP kinases for each of the Cdc42p-dependent MAP kinase pathways was also examined. To examine basal and activated conditions, cells were grown in media containing galactose over a time-course experiment, which induces phosphorylation of the fMAPK Kss1p [P∼Kss1p, (29, 65, 91)]. As has been reported (29, 87), the levels of P∼Kss1p increased in galactose over time (Fig. 3*D*, control, 1 h; 3 h). Overexpression of *BMH1* induced high levels of P∼Kss1p over the course of the experiment (Fig. 3*D*, *BMH1*, 0 min through 3 h). This result indicates that overproduction of Bmh1p induces the fMAPK pathway. Under inducing conditions (3 h), *BMH1* did not cause a further increase in P∼Kss1 levels above its maximal levels (Fig. 3*D*, graph, 3 h), which may indicate that Bmh1p preferentially impacts the basal-to-activated signaling of the fMAPK pathway.

A similar approach was used to evaluate the role of Bmh1p in regulating the HOG pathway. Phosphorylation of the MAPK Hog1p (P∼Hog1p) was assessed by growth of cells in salt, which caused a rapid increase in P∼Hog1p levels (Fig. 3*E*). Overexpression of *BMH1* did not impact P∼Hog1p levels at any time point tested (Fig. 3*E*, *BMH1*, 0 min). In fact, the level of P∼Hog1p was lower for several time points tested including no-salt environments (t = 0). Therefore, overexpression of Bmh1p might result in a slight reduction in HOG pathway activity.

**Figure 5:**
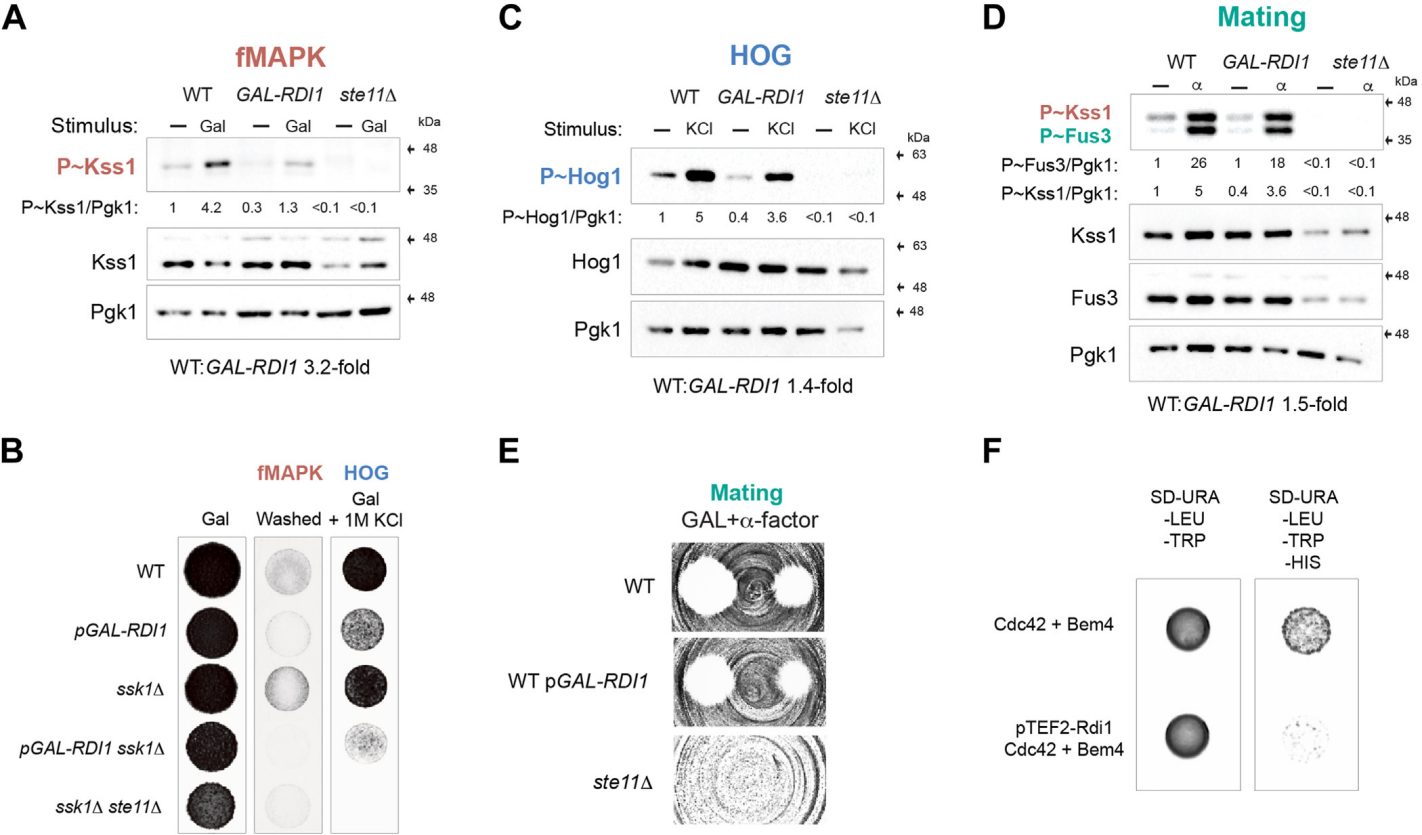
***RDI1* overexpression inhibits the activity of Cdc42-dependent MAPK pathways.***A*, WT cells or cells overexpressing *RDI1* and the *ste11*Δ mutant (all in the *ssk1*Δ background) were grown in YPD (−) or YP-GAL (Gal) media for 5.5 h at 30 °C. Immunoblots were probed with p44/42 antibodies to detect P∼Kss1p and P∼Fus3p, as well as α-Kss1p and α-Pgk1p antibodies as indicated. The P∼Kss1/Pgk1 ratio refers to relative levels of P∼Kss1p to Pgk1p of the blot shown. *B*, WT cells and the indicated mutants were grown in YP-GAL (Gal) and YP-GAL+1M KCl (Gal+1M KCl) media for 3 days at 30 °C. Plates were photographed, and the YP-Gal plate was washed in a stream of water and photographed again (washed). *C*, same cells described in panel *A* were grown in YP-GAL (−) or YP-GAL supplemented with 1M KCL (KCl) for 5 min at 30 °C. Immunoblots were probed with p38 antibodies to detect P∼Hog1p, α-Hog1p, and α-Pgk1p antibodies. P∼Hog1/Pgk1 ratio refers to relative levels of P∼Hog1p to Pgk1p of the blot shown. *D*, same cells described in panel *A* were grown in YP-GAL (−) or YP-GAL supplemented with 6 μM of α-factor (α) for 5 min at 30 °C. Immunoblots were probed with p44/42 antibodies to detect P∼Kss1p and P∼Fus3p, α-Kss1p, α-Fus3p, and α-Pgk1p antibodies. P∼Kss1/Pgk1 ratio refers to relative levels of P∼Kss1p to Pgk1p of the blot shown. P∼Fus3/Pgk1 ratio refers to relative levels of P∼Fus3p to Pgk1p of the blot shown. *E*, halo formation in response to α-factor of same cells described in panel *A*. Cells were spread on YP-GAL media and α-factor was spotted at two concentrations on the surface, 2 and 6 μM, to study cell-cycle arrest. *F*, yeast two-hybrid analysis between Cdc42p and Bem4p in WT cells and cells where the gene expression of *RDI1* controlled by the constitutive promoter *TEF2*.

In response to the mating pheromone α-factor (after 5 min), WT cells show ∼7-fold increase in P∼Fus3p (Fig. 3*G*, Control, compare 0 min to 5 min). Overexpression of *BMH1* did not cause an additional increase phosphorylation of Fus3p above the control (Figs. 3*F* and 5 min, compare control to *BMH1*) and did not affect the mating phenotype of shmoo formation (Fig. S3*B*). Overexpression of *BMH1* did show one difference from the control at 0 min, which was higher levels of P∼Fus3p (Figsures 3G and 0 min, compare control to *BMH1*). These results suggest that Bmh1p and Bmh2p do not regulate the mating pathway, except possibly for basal levels. Overall, these results show that Bmh1p and Bmh2p mainly regulate the Ste20p branch of the fMAPK pathway.

### Role for other Cdc42p-interacting proteins in regulating MAPK pathways

**Figure 4:**
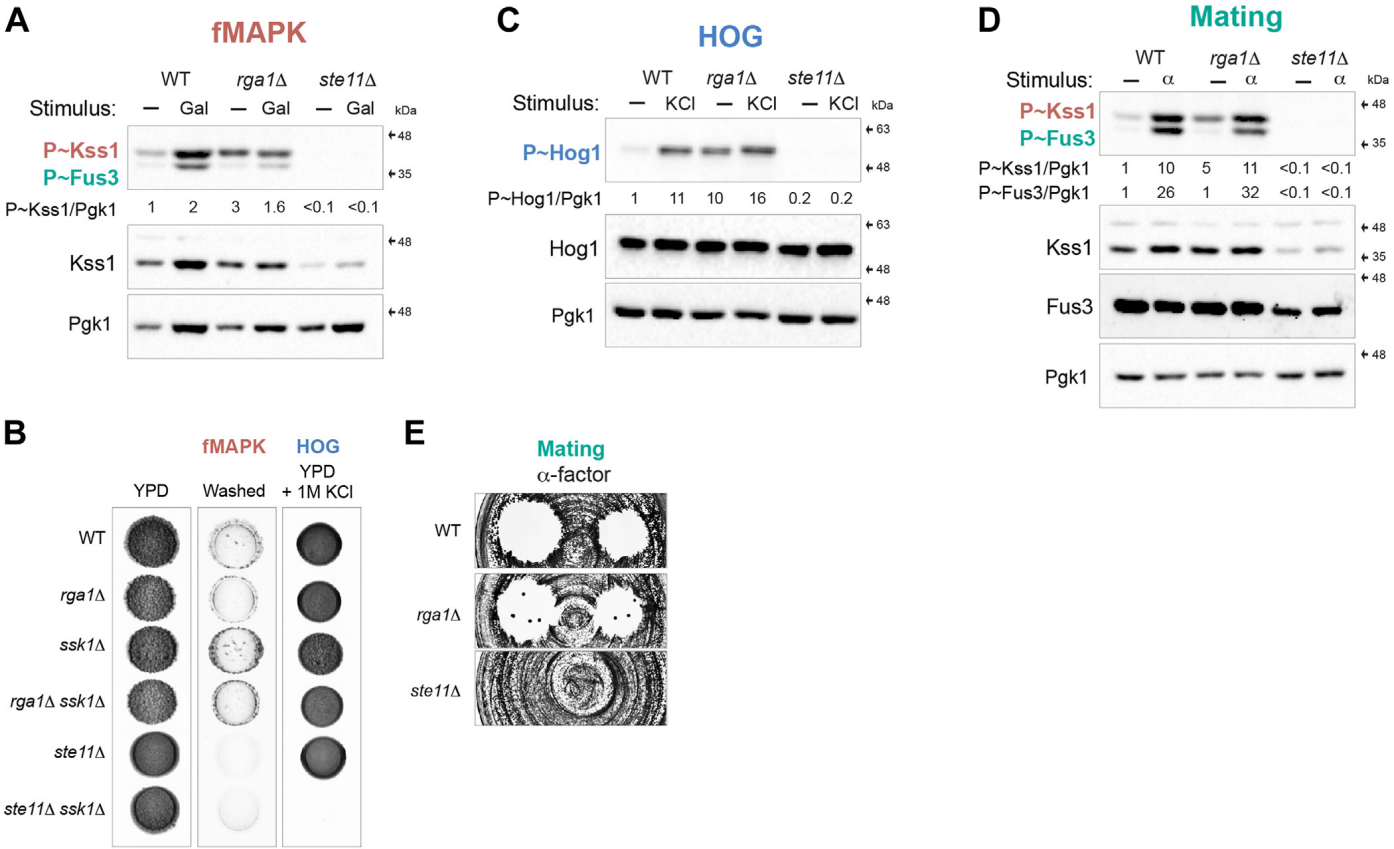
**Rga1p regulates basal activity of Cdc42-dependent MAPK pathways.***A*, WT cells (PC6810) and the *rga1*Δ (PC6687) and *ste11*Δ (PC6604) mutants form the *ssk1*Δ background were grown in YPD (−) or YPGAL (Gal) for 5.5 h at 30 °C. Immunoblots were probed with p44/42 antibodies to detect P∼Kss1p and P∼Fus3p, α-Kss1p and α-Pgk1p antibodies. P∼Kss1/Pgk1 ratio refers to relative levels of P∼Kss1p to Pgk1p of the blot shown. *B*, WT cells and mutants indicated were grown in YPD media for 3 days at 30 °C (YPD) and the plate was washed (fMAPK). Same cells were grown in YPD supplemented with 1M KCl for 3 days at 30 °C (HOG). *C*, same cells described in panel 4A were grown in YPD (−) or YPD supplemented with 1M KCL (KCl) for 5 min at 30 °C. Immunoblots were probed with p38 antibodies to detect P∼Hog1p, α-Hog1p, and α-Pgk1p antibodies. P∼Hog1/Pgk1 ratio refers to relative levels of P∼Hog1p to Pgk1p of the blot shown. *D*, same cells described in panel *A* were grown in YPD (−) or YPD supplemented with 6 μM of α-factor (α) for 5 min at 30 °C. Immunoblots were probed with p44/42 antibodies to detect P∼Kss1p and P∼Fus3p, α-Kss1p, α-Fus3p, and α-Pgk1p antibodies. P∼Kss1/Pgk1 ratio refers to relative levels of P∼Kss1p to Pgk1p of the blot shown. P∼Fus3/Pgk1 ratio refers to relative levels of P∼Fus3p to Pgk1p of the blot shown. *E*, halo formation in response to α-factor of same cells described in panel *B*. Cells were spread on YPD media and α-factor was spotted at two concentrations on the surface, 6 and 2 μM, to study cell-cycle arrest. MAPK, mitogen-activated protein kinase.

Rho GTPases are inactivated by GAPs that stimulate their intrinsic GTPase activity. Rga1p is the main GAP for Cdc42p in the fMAPK pathway (27), and as shown above, cells lacking Rga1p showed elevated levels of P∼Kss1p (Fig. 2*E*). To further explore this phenotype, the fMAPK pathway was examined under basal (glucose replete) and pathway-activated (galactose) conditions. In glucose-replete conditions, the *rga1*Δ mutant showed elevated levels of P∼Kss1p (Fig. 4*A*, minus sign refers to glucose-replete conditions, compare WT to *rga1*Δ). By comparison, Rga1p did not dampen fMAPK pathway activity under pathway-activating conditions (Fig. 4*A*, Gal, compare WT to *rga1*Δ). Cells lacking Rga1p also showed a similar pattern of invasive growth as WT (Fig. 4*B*). These results were surprising because Rga1p is expected to function under conditions when Cdc42p is active in the fMAPK pathway. Therefore, Rga1p regulates the fMAPK pathway under basal conditions but not in conditions when the pathway is active and cells are undergoing invasive growth.

Rga1p also dampened basal HOG pathway activity (Fig. 4*C*) but had a modest effect when cells were induced by osmotic stress (Fig. 4*C*, KCl). Similarly, Rga1p did not impact osmotolerance, as seen in cells lacking the redundant Sln1p branch of the HOG pathway (Fig. 4*B*, *ssk1*Δ). Cells lacking Rga1p also showed higher basal mating pathway activity (Fig. 4*D*) but did not impact P∼Fus3p levels after treatment with α-factor (Fig. 4*D*). Similarly, the growth arrest in response to α-factor was not impacted in cells lacking Rga1p (Fig. 4*E*). Therefore, Rga1p negatively regulates Cdc42p-dependent MAPK pathways mainly under basal conditions.

Rho GTPases are also regulated by Rho GDP-dissociation inhibitors which control their subcellular localization by sequestering GTPases from membranes (92, 93). Rdi1p is the sole guanine nucleotide dissociation inhibitor in yeast (94, 95) and was tested for roles in MAPK pathway regulation. As cells lacking *RDI1* had subtle phenotypes, the activity of MAPK pathways was examined in cells overexpressing *RDI1* by a galactose-inducible promoter. Overexpression of *RDI1* would be expected to promote Cdc42p extraction from membranes and cause a decrease in MAPK pathway activity. As expected, overexpression of *RDI1* caused a reduction in fMAPK pathway activity under basal (minus, glucose – cells were pre-grown in galactose to induce *RDI1* expression) and activating conditions (Gal) (Fig. 5*A*). *RDI1* overexpression also caused a reduction in invasive growth (Fig. 5*B*, washed). Overexpression of *RDI1* also dampened the HOG (Fig. 5*C*, 1.4-fold) and mating (Fig. 5*D*, 1.5-fold) pathways but to a lesser degree than fMAPK (Fig. 5*A*, 3.2-fold). The fMAPK pathway was more affected than the HOG and mating pathways by functional tests (Fig. 5, *B* and *E*). The interaction between Bem4p and Cdc42p is required for fMAPK pathway activation (29). Overexpression of *RDI1* by a galactose-inducible promoter reduced the interaction between Cdc42p and Bem4p by two-hybrid analysis (Fig. 5*F*). These results indicate that Cdc42p levels at the plasma membrane are critical for MAPK pathway signaling. Moreover, the activity of the fMAPK pathway is highly sensitive to Cdc42p levels at the plasma membrane.

**Figure S4:**
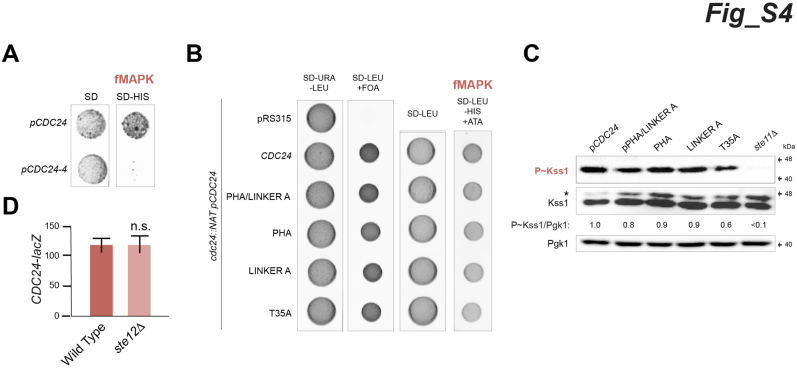


Rho GTPases are activated by GEFs that promote nucleotide exchange from GDP to GTP. Cdc24p is the main GEF for Cdc42p and is required for fMAPK pathway activity (28, 74). Cdc24p is phosphorylated in a cell cycle–dependent manner (96, 97, 98) in several domains of the protein (99). To determine whether phosphorylation of Cdc24p impacts the fMAPK pathway, versions of Cdc24p containing substitutions in known phosphorylation sites were examined in the conditional *cdc24-4* mutant, which is defective for fMAPK pathway activity (Fig. S4*A*). All of the phosphosite mutants tested (including a version lacking all 35 phosphorylation sites, T35A) rescued the viability defect of the *cdc24::NAT* mutant, based on growth on 5-floroorotic acid, and retained function in the fMAPK pathway based on *FUS1-HIS3* (Fig. S4*B*) and P∼Kss1p analysis (Fig. S4*C*). Therefore, the phosphorylation of Cdc24p is not critical for regulation of the fMAPK pathway. In *Candida albicans*, expression of the *CDC24* gene is induced during hyphal growth and is a transcriptional target of the homologous Cek MAPK pathway (100). However, *CDC24* expression was not dependent on the fMAPK pathway, based on a transcriptional reporter expression profiling data from our laboratory (101, 102, 103), and because overexpression of *CDC24* does not induce fMAPK pathway activity or invasive growth in *S. cerevisiae* (87). Thus, phosphorylation of Cdc24p and transcriptional induction of the *CDC24* gene are not major determinants in the regulation of the fMAPK pathway.

## Discussion

**Figure 6:**
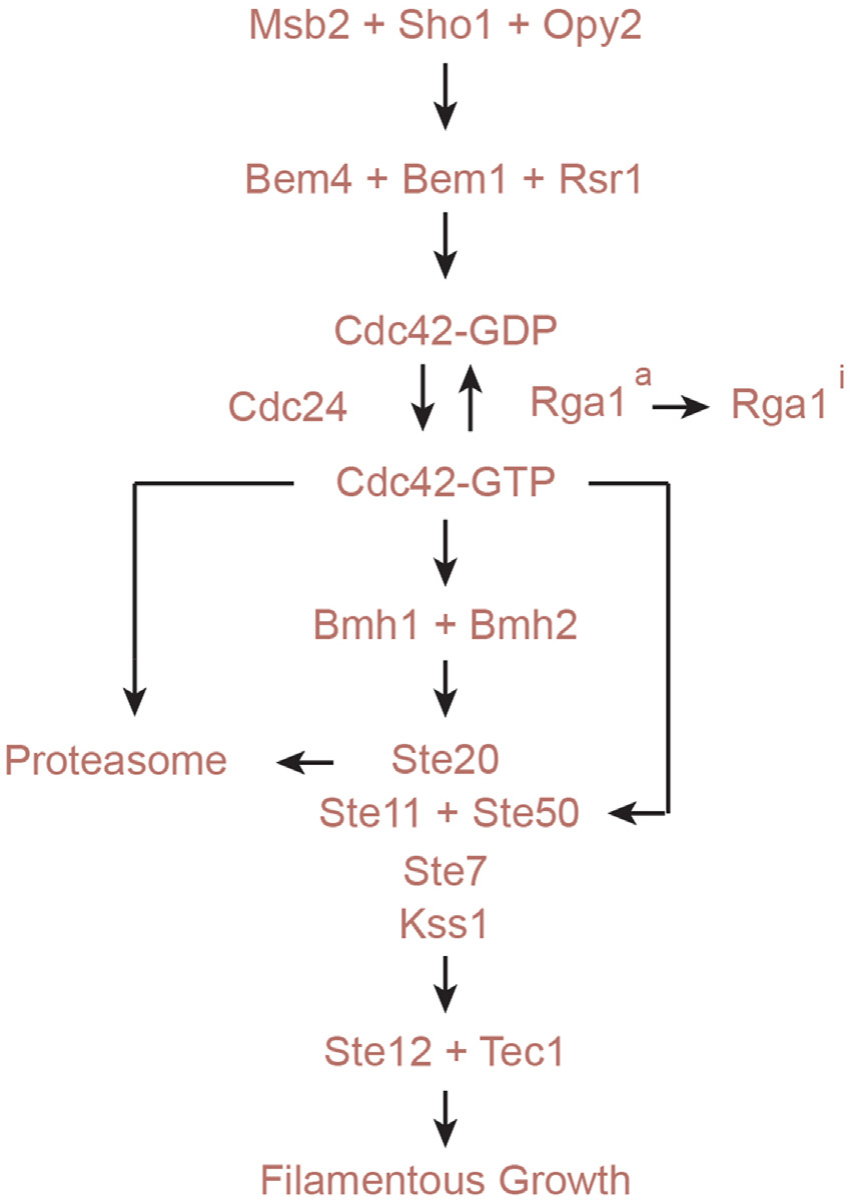
**Updated model for the regulation of the fMAPK pathway.** Msb2p and Sho1p sensors connect to the GTPase module through Bem-type adaptors. Cdc42p and Ste20p are turned over in the proteasome. Rga1p inhibits basal fMAPK pathway activity (Rga1^a^, a for active) but is inhibited in some manner to prevent its inhibitory effects on an activated pathway (Rga1^i^, i for inhibited). Cdc42p induces Ste20p-dependent and Ste20p-independent branches to control fMAPK pathway activity and filamentous growth.

Signaling pathways regulate biological responses by protein modules that are highly conserved structurally and functionally from yeast to humans. MAPK pathways can be regulated by sensor proteins that connect to and regulate GTPase modules. By characterization of these modules and understanding how they connect to and regulate one another, new insights into the regulation of biological processes can be appreciated. These include studies on regulated turnover, positive feedback, and the relationship between basal and activated states (104). Here, we report new aspects of the regulation of a Cdc42p-dependent MAPK pathway in yeast (Fig. 6) that may extend to MAPK pathway regulation in other systems.

### Interaction with Cdc42p is required for stabilization of the PAK Ste20p

A canonical aspect of MAPK pathway regulation involves the interaction with Rho GTPases, like Cdc42p, with PAK kinases (105). In yeast, Cdc42p interacts with the PAK Ste20p to induce the activation of MAPK pathways (35, 66). Here, we identify a new feature of PAK regulation in yeast, by turnover of the Ste20p protein in the 26S proteasome. In mammals, this regulatory feature has also been defined (106) and may represent an evolutionarily conserved way to modulate the activity of this type of protein kinase. In the amino-terminal domain of Ste20p, amino acid residues 333 to 370 (CRIB domain) are responsible for binding to the active or GTP-bound conformation of Cdc42p. We found that Ste20p lacking its CRIB domain is not stable and is turned over by the 26S proteasome. These results indicate that Cdc42p may protect Ste20p from turnover by the proteasome. This hypothesis was corroborated by examination of a version of Cdc42p that is defective for interaction with Ste20p, which also showed reduced levels of Ste20p in the cell. It is possible that Ste20p that cannot bind to Cdc42p is unstable and is rapidly turned over as part of a quality-control mechanism. In this case, Cdc42p may function to fold or otherwise stabilize the Ste20p protein. Alternatively, Cdc42p may occlude recognition sites in the N terminus of Ste20p by ubiquitin ligases that promote turnover of the protein.

Ste20p may be degraded in a regulated manner through its interaction with Cdc42p (see the model in Fig. S5). We have previously shown that Cdc42p is turned over, which occurs preferentially when the protein is in the GTP-bound state (65). Here, we show that Ste20p is protected by GTP-bound Cdc42p. These findings indicate that GTP-bound Cdc42p may be degraded, which leads to turnover of Ste20p when no longer bound to the GTPase. In this way, cells may achieve a more permanent attenuation of MAPK pathways. Ste20p levels may be regulated through the cell cycle, as the protein has a specific function at preinitiation sites early in G1 (68) where it is recruited to the plasma membrane when bound to GTP-Cdc42p (66). Overall, our findings surrounding the stability of a Rho GTPase and PAK kinase add a new layer of regulation to MAPK pathway signaling.

### A Ste20p-independent branch of the fMAPK pathway

As studies of signaling pathways become more nuanced, it is becoming increasingly clear that pathways are not strictly linear modules but contain ‘branches’ that function with specific proteins and/or in distinct settings. One function of such branches is to allow signals to become integrated into a pathway’s activity (107). For example, RAS/PKA and TOR converge on a common set of target genes (108). A second example comes from the HOG pathway, which is composed of two branches (Sln1p and Ste11p) that converge on the MAPKK Pbs2p (49). Within the Ste11p branch, two mucins function in distinct sub-branches converging on the tetraspan protein Sho1p (109). Furthermore, Ste20p and another PAK kinase, Cla4p, constitute sub-branches of the Ste11p branch of the HOG pathway (110, 111). While the logic of such extensive branching remains to be revealed, this knowledge helps account for the versatility of signaling pathways, which is probably a universal feature of signaling modules. By examining a newly characterized hyperactive version of Cdc42p, we identified a branch of the fMAPK pathway that does not require Ste20p (Fig. 6). This result was independently verified by examining cells lacking one of the GAPs for Cdc42p, Rga1p, which also showed Ste20p-independent activity. Like for the HOG pathway, Cdc42p might also regulate the fMAPK pathway through Cla4p. Alternatively, Cdc42p might directly activate Ste11p. In this scenario, Cdc42p would function analogously to the GTPase Ras in humans, which directly interacts with the MAPKKK, Raf. We found that the adaptor protein Bem4p, which interacts with both Cdc42p and Ste11p, was required for Ste20p-independent signaling, which might provide a connection between the two proteins. Bem4p might regulate pathway activity in cells lacking Ste20p due to its role in protecting Cdc42p; however, Cdc42p^Q61L+TD^ can bypass Bem4p in the fMAPK pathway, which identifies a potentially new function for Bem4p as a conduit between Cdc42p with Ste11p. The Ste20p-independent branch might amplify MAPK pathway activity in certain circumstances or sensitize the pathway to specific inputs. For example, ethanol stimulates filamentous growth by a mechanism that requires Ste11p but not Ste20p (112).

### Cdc42p-dependent MAPK pathways function differently in basal and activating conditions

We examined proteins that interact with Cdc42p to define their roles in MAPK pathway regulation. We confirmed that the 14-3-3 proteins regulate the fMAPK pathway, and as expected from previous findings (64), they function in Ste20p-dependent signaling (Fig. 6). Interestingly, when overexpressed, the Bmh proteins did not stimulate all three Cdc42p-dependent MAPK pathways but rather (modestly) dampened basal HOG and mating activity. There may be a pathway-specific association between the Bmh proteins and MAPK pathway-specific regulators, such as Bem4p. Recently, Bmh1p and Bmh2p have been identified as key regulators of meiotic commitment in yeast (113). These proteins also impact retrograde mitochondria-to-nucleus signaling (114) and other pathways such as the target of rapamycin or TOR pathway (115). Given that filamentous growth occurs differently in haploids and diploids (14) and is impacted by the retrograde (116) and TOR pathways (117), the 14-3-3 proteins might impact filamentous growth and signaling at multiple levels.

We also found that Rga1p, the main GAP for Cdc42p in the fMAPK pathway, exerts its effect on Cdc42p mainly in the basal state (Fig. 6). How is Rga1p restricted from attenuating active MAPK pathways? Rga1p may get evicted from complexes containing active Cdc42p. It may be excluded by PAK binding to Cdc42p or modified by a posttranslational modification to inactive its GAP activity. Rga1p′s localization is critical for its function (118), and it may be that its localization is altered in response to MAPK pathway activation. Rga1p is important for attenuating basal signaling, while proteasomal Cdc42p degradation may regulate activated signaling. Perhaps another GAP is responsible for attenuating activated signaling. This idea is not supported by phenotypic analysis because other GAPs do not show phenotypes in filamentous growth. This idea is also not supported by genetic screens for negative regulators of the fMAPK pathway, which have typically identified a single GAP, Rga1p.

Our results also identify distinctions between the ways that signaling pathways are regulated in basal and activated states. We have previously shown that bud-site selection proteins regulate the fMAPK pathway, and their roles are more critical during basal signaling (119). Rga1p may be more effective at dampening the fMAPK through a mechanism involving bud-site selection proteins. Indeed, Rga1p is critical for preventing budding at previous bud sites (83, 120, 121).

We also show that there are higher levels of phosphorylated Kss1p than phosphorylated Fus3p under basal conditions. This might be because Fus3p is conformationally constrained (locked) such that it is only activated in the presence of pheromone (61). These results imply that cells may be more potentiated for filamentous growth than for mating, even under nutrient-replete conditions. In line with this idea, cells under nutrient-replete conditions exhibit some fMAPK-dependent phenotypes, such as cell adhesion (28).

## Experimental procedures

### Yeast strains, reagents, and media

Strains are listed in Table 1, and plasmids are listed in Table 2. Yeast were grown in synthetic media (SD; 0.67% yeast nitrogen base without amino acids, 2% dextrose), supplemented with amino acids as required, yeast extract peptone dextrose media (1% bacto-yeast extract, 2% bacto-peptone, 2% dextrose) and YEPGAL (1% bacto-yeast extract, 2% bacto-peptone, 2% galactose) at 30 °C. Gene disruptions were performed by antibiotic resistance markers *NAT*, *HYG*, and *KanMX6* using PCR-based approaches using published templates (122, 123). Yeast strains and plasmids have been previously described. Some gene deletions were constructed using cassettes that contained antibiotic resistance markers. Plasmid pRL116 CEN/URA4 pGFP-Ste20p and pGFP-Ste20p-CRIBΔ were provided by P. Pryciak (35). Plasmids pRS315 and pRS316 have been described (124). The Cdc24p phosphorylation sites WT (PC3622) and mutants including Cdc24^PH-A^ (PC3624), Cdc24^Linker-A^ (PC3625), Cdc24^PH-A, Linker-A^ (PC3623), and Cdc24^T35A^ (PC3626) were generously shared by Rong Li (125). Plasmids carrying Bem1p-12XMYC (pDLB2374) were provided by Daniel Lew (34). Two-hybrid analysis was performed as described in (29). Tests for viability and function of *CDC24* alleles were performed by plasmid loss experiments of the pCDC24-based URA3 plasmid on 5-floroorotic acid (126). pCDC24-lacZ (PC2107) was constructed by *in vivo* recombination of the PCR-amplified *CDC24* gene into vector V84 provided by C. Boone.

**Table 1:** Yeast strains used in the study

Name	Genotype	Reference
PC313^a^	*MAT***a***ura3-52*	(40)
PC538^a^	*MAT***a***ste4 FUS1-lacZ FUS1-HIS3 ura3-52*	(17)
PC673^a^	*MAT***a***ste4 FUS1-lacZ FUS1-HIS3 ura3-52 ste20::kanMX6*	(17)
PC986^b^	*MAT*α *his3Δ0, leu2Δ0, met15Δ0, ura3Δ0*	(131)
PC1894^a^	*MAT***a***ste4 FUS1-lacZ FUS1-HIS3 ura3-52 leu2::HYG*	(28)
PC2382	*MAT***a***ste4 FUS1-lacZ FUS1-HIS3 ura3-52 ste12::kanMX*	(28)
PC3391^a^	*MAT***a***ste4 FUS1-lacZ FUS1-HIS3 ura3-52 rga1::NAT*	(28)
PC3392^a^	*MAT***a***ste4 FUS1-lacZ FUS1-HIS3 ura3-52 ste12::kanMX6 rga1::NAT*	(28)
PC3393^a^	*MAT***a***ste4 FUS1-lacZ FUS1-HIS3 ura3-52 bem4::HYG rga1::NAT*	(28)
PC3551^a^	*MAT***a***ste4 FUS1-lacZ FUS1-HIS3 ura3-52 leu2::HYG bem4::NAT*	(28)
PC3862^a^	*MAT***a***ste4 FUS1-lacZ FUS1-HIS3 ura3-52 ste11::NAT*	(28)
PC5851^b^	*MAT***a***ura3-52 his3-200 ade2-101 his3-200 leu2-1*	(70)
PC5852^b^	*MAT***a***ura3-52 his3-200 ade2-101 his3-200 leu2-1 cim3-1*	(70)
PC5024^a^	*MAT***a***ura3-52 ste11::NAT*	(28)
PC6016^a^^,^^c^	*MAT***a***can1*Δ::*Ste2pr-spHIS5 lyp1*Δ::*Ste3pr-LEU2 his3*::*hisG* l*eu2*Δ*0 ura3*Δ*0*	(132)
PC6102^a^	*MAT***a***ste4 FUS1-lacZ FUS1-HIS3 ura3-52 tec1::NAT*	(101)
PC6539^a^	*MAT***a***ste4 FUS1-lacZ FUS1-HIS3 ura3-52 cdc42::NAT* pRS316-GFP-linker-CDC42	(65)
PC6591^a^	*MAT***a***ura3-5 leu2*	(65)
PC6810^a^^,^^d^	*MAT***a***ura3-52 leu2 ssk1*	(29)
PC6604^a^	*MAT***a***ura3-52 leu2 ssk1 ste11::NAT*	(29)
PC6684^a^	*MAT***a***ura3-52 leu2 ssk1 cdc42::NAT* pRS316-GFP-linker-CDC42	(29)
PC6687^a^	*MAT***a***ura3-52 leu2 ssk1 rga1::NAT*	(29)
PC6936^a^	*MAT***a***ura3-52 cdc24::NAT leu2::HYG pRS315 cdc24-4*	(74)
PC7501^a^	*MAT***a***ura3-52 cdc24::NAT leu2::HYG pRS315 cdc24-4* pRS316	This study
PC7772^a^	*MAT***a***ura3-52 leu2 ssk1 ste20::NAT*	This study
PC7777^a^	*MAT***a***ste4 FUS1-lacZ FUS1-HIS3 ste20::NAT rga1::HYG*	This study
PC7780^a^	*MAT***a***ste4 FUS1-lacZ FUS1-HIS3 ste20::NAT bem4::HYG*	This study
PC7886^a^	*MAT***a***ste4 FUS1-lacZ FUS1-HIS3 bem4::HYG rga1::NAT ste20::GENT*	This study

Bold indicates that it is a historical designation for this mating type nomenclature in yeast.

a Strains are Σ1278b background.

b S288c background.

c Strains from an ordered deletion collection described in (132) were also used.

d Strains containing three *CDC42* alleles (K5A, V36T, and F37Y) were constructed in the Σ1278b background and described in (29).

**Table 2:** Plasmids used in the study

Name	Description	Reference
PC1441	YCp50-STE11-4	(133)
PC1696	pMsb2-GFP	(85)
PC1715	pRS316-SHO1(P120L)-GFP	(85)
PC2150	pRS316-CDC24	(28)
PC1422	pRS315	(124)
PC2769	pV84	(134)
PC2107	p*CDC24-lacZ*	This study
PC2207	pRS316	(124)
PC4394	pRL116 CEN/URA3 GFP-Ste20	(35)
PC4395	pRL116 CEN/URA3 GFP-Ste20^334-369Δ (CRIBΔ)^	(35)
PC6455	pRS306-GFP-linker-CDC42 (pDLB3609)	(34)
PC6454	pRS316-GFP-linker-CDC42	(29)
PC6457	pRS315-GFP-linker-CDC42	(29)
PC7458	pRS316-GFP-linker-CDC42 (Q61L)	(65)
PC7654	pRS316-GFP-linker-CDC42 (Q61L+ TD: K5R, Q61L, K94R, K96R)	(65)
PC3621	pSW17 control plasmid	(99)
PC3622	pSW72 *CDC24*	(99)
PC3623	pSW76 *CDC24* with PH-A and Linker A mutations	(99)
PC3624	pSW78 *CDC24* with PH-A mutations	(99)
PC3625	pSW80 *CDC24* with Linker A mutations	(99)
PC3626	pSW82 *CDC24* with T35A mutations	(99)
PC7670^a^	pRS316-GFP-linker-CDC42 (V36T)	This study

a The pBMH1 and pBMH2 plasmids are driven by the *GAL1* promoter and have been described in (90).

### Immunoblot and phosphoblot analysis

Cells were grown to saturation in SD or yeast extract peptone dextrose media for 16 h and transferred fresh media and grown for 4 to 6 h to mid log phase. Cells were harvested by centrifugation. Proteins extracts were prepared by mechanical disruption with beads followed by a trichloroacetic acid precipitation method (29). Protein precipitates were analyzed by SDS-PAGE and transferred to a nitrocellulose membrane (Cat#10600003, Amersham Protran Premium 0.45 μm NC, GE Healthcare Life sciences). Monoclonal mouse anti-GFP antibodies were used (Cat#11814460001, clones 7.1 and 13.1, Roche) at 1:1000 dilution. Polyclonal rabbit phospho-p44/42 MAPK (Erk1/2, Cat#3102, Cell Signaling Technology) and p38 (Cat#9211, Cell Signaling Technology) were used at 1:10,000 dilution. Mouse anti-Kss1p antibodies (yC-19, Santa Cruz Biotechnology) and anti-Hog1p antibodies (Cat# yC-20, Santa Cruz Biotechnology) were used at 1:10,000 dilution. Monoclonal mouse anti-Pgk1p antibodies (22C5D8, Cat#459250, Invitrogen) were used at 1:1000 dilution. Secondary anti-mouse IgG-HRP (Cat# 1706516, Bio-Rad Laboratories) and goat anti-rabbit IgG-HRP (Cat#115-035-003, Jackson ImmnunoResearch Laboratories) were used. The nitrocellulose membrane was blocked with 5% non-fat dried milk or 5% bovine serum albumin (for p44/42 antibody) for 1 h prior antibody detection. Primary incubations were performed at 4 °C for 16 h and secondary at 20 °C for 1 h. Immunoblots were visualized by the Gel Documentation XR Imaging System (Bio-Rad, Inc), after addition of Chemiluminescent HRP substrate for chemiluminescent Westerns (Radiance Plus Substrate, Azure Biosystems).

Band intensities quantitation of P∼Fus3p, P∼Kss1p, Cdc42p and GFP-Cdc42p, and ubiquitin were detected under nonsaturated conditions and normalized to the housekeeping protein Pgk1p using the Image Lab Software (https://www.bio-rad.com/en-us/product/image-lab-software?ID=KRE6P5E8Z; Bio-Rad, Inc). WT cells and control conditions were set to a value of one and adjusted for other samples accordingly.

### MAPK pathway reporters and functional tests

The fMAPK pathway activity was evaluated by assessing the *FUS1-HIS3* growth reporter in cells lacking *STE4* (19, 127). Cells lacking an intact mating pathway (*ste4*Δ), show basal activity of the fMAPK pathway by this reporter (17). WT cells (PC538) and a control strain (*ste11*Δ, PC3862) were grown in SD-URA media to maintain plasmid selection (SD-URA-HIS, control) and media lacking histidine (SD-URA-HIS) or supplemented with 3-amino-1,2,4-triazole to evaluate fMAPK activity.

The *FRE-lacZ* (41) and *FUS1-LacZ* (*ste4* background) (101) reporters were also used to study the fMAPK pathway activity. The plate washing was performed as described in (14) to evaluate the fMAPK pathway phenotype. To evaluate the HOG pathway, cells were grown in media supplemented with 0.5 M KCl or 1 M KCl and analyzed by phophoblot analysis or by growth on semi-solid agar media (128). Cells lacking the Sln1p branch of the HOG pathway (*ssk1*Δ) were examined, which is redundant with the Ste11p branch (49). The mating pathway activity was evaluated by phosphoblot analysis, halo assays, and shmoo formation by microscopy (129). For halo assays, cells were spread on SD-URA or YPD semi-solid agar media. After the liquid had evaporated, alpha factor was applied to the surface of the plate, which was incubated for 2 days at 30 °C.

### Fluorescence microscopy

Differential interference contrast, fluorescence microscopy using FITC, and rhodamine filter sets were used in an Axioplan 2 fluorescence microscope (Zeiss) with a Plan-Apochromat 100x/1.4 (oil) objective with the Axiocam MRm camera (Zeiss). Images were analyzed using Axiovision 4.4 software (https://www.zeiss.com/microscopy/en/service-support/support/discontinued-products.html; Zeiss). Actin stanning was performed as described (130) using Phalloidin-Atto 532 (Millpore Sigma, 49,429). Images were analyzed in Adobe Photoshop and ImageJ (https://imagej.nih.gov/ij/download.html). Fluorescence images were converted to Grayscale and inverted using ImageJ.

### Statistical analysis

All statistical tests were performed with Prism 7 (GraphPad; https://www.graphpad.com/scientific-software/prism). Data were analyzed by one-way ANOVA test followed by a Tukey’s multiple comparison test to generate *p*-values.

## Data availability

All the data are included in the manuscript and supplemental files and are available upon request at pjcullen@buffalo.edu.

## Supporting information

This article contains supporting information.

## Figures and Tables

**Figure S5:**